# Gender-related data missingness, imbalance and bias in global health surveys

**DOI:** 10.1136/bmjgh-2021-007405

**Published:** 2021-11-26

**Authors:** Ann M Weber, Ribhav Gupta, Safa Abdalla, Beniamino Cislaghi, Valerie Meausoone, Gary L Darmstadt

**Affiliations:** 1 School of Public Health, University of Nevada Reno, Reno, Nevada, USA; 2 Department of Medicine, University of Minnesota School of Medicine, Minneapolis, Minnesota, USA; 3 Global Center for Gender Equality, Department of Pediatrics, Stanford University School of Medicine, Stanford, California, USA; 4 Department of Global Health and Development, London School of Hygiene and Tropical Medicine, London, UK; 5 Stanford Research Computing Center, Stanford University, Stanford, California, USA

**Keywords:** health policy, public health, epidemiology

## Abstract

Global surveys have built-in gender-related biases associated with data missingness across the gender dimensions of people’s lives, imbalanced or incomplete representation of population groups, and biased ways in which gender information is elicited and used. While increasing focus is being placed on the integration of sex-disaggregated statistics into national programmes and on understanding effects of gender-based disparities on the health of all people, the data necessary for elucidating underlying causes of gender disparities and designing effective intervention programmes continue to be lacking. Approaches exist, however, that can reasonably address some shortcomings, such as separating questions of gender identification from biological sex. Qualitative research can elucidate ways to rephrase questions and translate gendered terms to avoid perpetuating historical gender biases and prompting biased responses. Non-health disciplines may offer lessons in collecting gender-related data. Ultimately, multidisciplinary global collaborations are needed to advance this evolving field and to set standards for how we measure gender in all its forms.

**Summary box**

- Current recommendations for improving gender-related data in global surveys fail to address the conflation of gender and sex; missing information for men, boys and gender minorities; or biases in question construction, survey translation or adaptation.
- We highlight existing approaches used in survey design to help address misclassification of gender identification, sex-imbalanced sampling, unintentional gender bias in question phrasing and translation, and missing or omitted data by sex and for a range of gender facets of people’s lives, including gender norms.
- Further research, including qualitative investigation, is needed to systematically identify and challenge instances of gender-related biases in the design of population-based surveys, and, once identified, to develop and test solutions.
- Multidisciplinary global collaborations are needed to resolve sources of gender-related data missingness, imbalance and bias in global health surveys if we are to use these data sources to further our understanding of gender-based disparities in health and well-being.

## Introduction

Existing global health surveys have important limitations for the study of gender and pathways linking gender to health inequities. We use illustrative (but not unique) survey examples to highlight six shortcomings associated with data ‘missingness’ in gender-related dimensions of people’s lives, imbalanced or incomplete representation of population groups, and biases in how gender information is ascertained. We highlight existing approaches to resolve some shortcomings and advocate for qualitative research and multidisciplinary global collaborations to find new ways to study the gender dimensions of people’s lives across diverse contexts.

## Missing information on gender identity and gendered experiences

Gender influences people’s opportunities, choices and health in a multitude of ways. Gender identity—people’s personal sense of being woman, man, non-binary or any other gender identity—can affect how one experiences life in a given society. Gender norms are the expectations of appropriate actions for people of each gender. They create an order that apportions power, resources, roles and social status on the basis of whether one is perceived of a given gender,^1^ even impacting one’s interactions with the health system.^2^

In previous research, we identified 17 publicly available population-based surveys that contained both health and gender-related indicators to examine their associations.^3^ These included the Demographic and Health Surveys (DHS), composed of >400 household surveys of >90 low-income and middle-income countries (LMICs), and the World Values Survey (WVS), an individual-level survey administered in nearly 100 countries at all income levels. In all identified surveys, a single binary indicator was available for sex of respondents (ie, male/female) with no option for intersex. Some surveys conflated sex and gender identity in a single question (ie, asking about gender with a binary response option of male/female), while others relied on interviewer observation to identify sex.^3^ Information on gender identity and facets of individuals’ gendered experiences was partially or entirely missing.

Such muddling and missingness are unsurprising, given that few validated measures of gender-related constructs exist.^4^ However, by not ascertaining an individual’s gender identity in population-level surveys, we fail to recognise and address health disparities of certain groups of people such as those identifying as genderqueer, gender non-conforming or gender fluid. Moreover, in failing to measure socially constructed gender roles, practices and relationship dynamics, we resort to categorising differences in health based on sex alone. Due to this lack of granularity in the data, researchers typically resort to using sex-disaggregated data, struggling to explain what is ultimately an undefined mix of health differences due to biological factors associated with sex and health disparities due to gender and its intersections with other social determinants of health.^5^

Importantly, other surveys have shown promising advances in assessing gender identity.^4 6^ Some researchers attempt to disentangle the sex that respondents were assigned at birth from the gender identity they align with most closely at the time of the survey, asking ‘What was your sex at birth? Female, male, intersex, prefer not to state,’ followed by ‘What is your current gender? Woman, man, non-binary, genderqueer, a gender identity not listed, prefer not to state.’ Recognising that categorisations of gender identity are imperfect and evolving,^7^ such an approach is practical, avoids a critical source of misclassification from relying on a two-category or three-category indicator for sex, and can uncover otherwise hidden health disparities.^8^ These questions may elicit varied or confused respondent reactions in some settings that require engagement of local stakeholders and qualitative research to carefully address. However, studying the experiences of transgender and gender diverse people across diverse sociocultural settings offers an opportunity to form a practical toolkit of appropriate questions about gender in global surveys. Quantifying the role of gender as a determinant of health disparities in the context of health differences due to sex is an ongoing grand challenge in global health.^3^

## Imbalanced and incomplete sample representation by sex and age

Reflecting its roots in reproductive health, the DHS often under-represents men and systematically omits all but minimal demographic information on children ages 6–14 years, and females and males over 49 and 59 years, respectively. The DHS emerged in 1984 as a follow-up to the World Fertility and Contraceptive Prevalence Surveys,^9^ designed to collect population-based information on reproductive health, fertility and under-5 child health and nutrition—issues historically viewed as women’s domains. Thus, DHS respondents were initially females of reproductive age (15–49 years). The men’s survey was introduced in 1987^10^ and, increasingly, males (15–59 years) are interviewed, but often in only half or a third of sampled households (purportedly for cost reasons) or, in some countries, not at all.^9^ Furthermore, nearly 13% of the DHS men’s surveys from 1985 to 2015 required males to be ever-married, currently married or married to the sampled female respondent, rendering some men’s health and health behaviours invisible in certain settings.^11^

The cost of obtaining data from a population-representative and balanced sample should be weighed against the implications of under-representing men or certain age groups. In simulations of cross-gender analyses (eg, estimating the influence of discordance in adult men’s attitudes and behaviours regarding premarital sex on adolescent women’s HIV risk) in the Zambia DHS, we demonstrated that imbalanced sampling by age and sex can lead to highly variable results, for example, in estimating risk for HIV infection, masking our ability to draw reliable insights and potentially misinforming programme design for both women and men.^12^ Although analytical methods exist to statistically rebalance data into a ‘representative’ population,^13^ these methods rely on assumptions that may be untestable. Ultimately, collecting balanced data on people across gender, sex and age groups will enable more accurate conclusions to be drawn on the influence of gender in the health and development of all people.

In contrast to the DHS, the World Bank’s (WB) high-frequency telephone surveys designed to monitor the poverty impact of COVID-19^14^ in >100 LMICs are an emerging source of data that is at risk of under-representing women. The WB surveys rely on a patchwork of sampling frames—including government registries, mobile phone ownership lists and contact telephone information from national household surveys—which favour men. Women are less likely to own a mobile phone in LMICs^15^ and recontact information for household surveys is often for the household head, most of whom are men.^16^ In Nigeria, women represented only 27% of respondents in the first wave of the WB phone survey^17^ and <20% of respondents in India.^18^ Notably, imbalances in the sex of respondents in these two country surveys are much greater than can be explained by male–female differences in mobile phone ownership (6% in Nigeria and 16% in India in 2020),^15^ suggesting that the practice of prioritising the household head for household surveys in these settings may be driving additional imbalance. In the most recent DHS surveys, 82% and 85% of households were male headed in Nigeria and India, respectively.^16^ Headship is defined as the person who ‘may be acknowledged as the head on the basis of age (older), sex (often, but not necessarily, male), economic status (main provider) or some other reason.’^19^ Although use of this definition of headship has been critiqued as patriarchal, inadequate and inappropriate,^20^ it continues, such that the self-identified household head is neither representative of the male or female adult population. To minimise non-representative sampling from household surveys, a WB tip sheet suggests randomly selecting a respondent from a household roster, unconditional on phone ownership, and, if necessary, asking for the phone to be passed to the randomly selected person.^21^ For random digit dialling, the tip sheets suggests that for one third of phone numbers, if a man answers the phone, interviewers ask to speak to an adult woman in the household either by passing the phone or calling back on the same or another number. Employing this last approach, nearly 50% of respondents in the WB’s first round of the Sierra Leone COVID-19 survey were women,^21^ despite an estimated 40% of women in Sierra Leone owning a mobile phone. We commend the WB for their quick action in filling a gap in knowledge of COVID-19 impacts. However, purposeful efforts are needed to apply lessons learnt, such as those in Sierra Leone, to assure proper representation of women and men impacted by the pandemic’s or any other health system disruption.

## Missing information by sex of respondent

**Figure 1:**
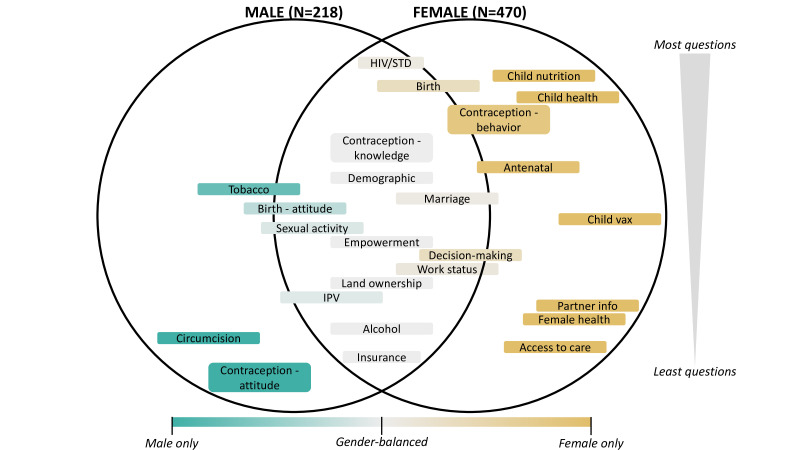
Topic areas included in the male and female modules of the phase 8 DHS questionnaire. Sex-specific topic areas are depicted by colour (teal for males and tan for females) with the depth of colour indicating the degree to which questions are unique to one sex. Questions were considered matched in both modules if they were identical or nearly identical (eg, the word ‘husband’ in the female module is replaced with the word ‘wife’ in the male module). Topics in light grey are covered equally in both modules. Topics that cross the lines are neither entirely unique nor equally matched by sex. Topics are arranged by total question count, with the largest topic areas at the top of the circles. DHS, Demographic and Health Surveys. IPV, Intimate Partner Violence; STD, Sexually Transmitted Disease.

Further echoing its history, topics covered in the DHS that were considered the explicit domain of women or men are often not explored with respondents of a different sex. To assess the current extent of such differential information gathering, we compared all questions in the male and female modules of the most recent DHS (Phase 8).^22^ We identified questions as either matched in modules for both men and women or unique to one sex, categorised them topically, and displayed the topic distribution in figure 1. Topics unique to females (about 65% of the female module) include questions about child health and nutrition, women’s health (eg, menstruation), partner demographic information, and factors influencing access to healthcare. While the male module has grown to 218 questions in phase 8, it has fewer than half the questions of the female module. About 25% of questions in the phase 8 male module are unique, including questions on circumcision and some questions on tobacco use.

Questions commonly used as proxies for women’s empowerment (eg, mobile phone ownership, land ownership, attitudes on wife beating) are asked in both modules, suggesting strong interest from the research community in a gender-balanced perspective on these topics. However, questions related to contraception are not fully matched: knowledge questions are asked of both sexes equally but 50 behaviour questions are asked of females and only five are asked of males. Further, two attitude questions are only asked of males: ‘women who use contraception may become promiscuous‘ and ‘contraception is a woman’s concern’, an attitude reflected in the larger number of contraception-related behaviour questions asked of women.

Overall, current DHS questionnaires ensure data on many important topics are collected from both men and women. Many of the sex-specific questions can be explained by biological differences (eg, questions about circumcision and cervical cancer) or as attempt to minimise redundancy (ie, asking female and her partner the same child questions). However, male care-seeking behaviour and participation in parenting, for example, could be assessed to capture non-gender normative practices and monitor change over time.

## Missing information on gender norms

Gender norms—the unwritten rules that govern acceptable actions for people of different genders—have important influences on health,^3^ but most research relies on proxies for gender norms such as social aggregates of individual behaviours or attitudes.^23^ Effective measurement of norms requires measuring: (1) empirical expectations (what respondents believe others are doing), (2) normative expectations (what respondents believe others expect them to do), (3) sanctions (reactions to their compliance or lack thereof) and (4) whose opinion matters to respondents and/or who governs sanctions (ie, the ‘reference’ group).^24^

Questions on gender norms need to be topic-specific and relevant to the context, requiring collaboration with local experts, and challenging their use in large-scale or multinational surveys. However, researchers are increasingly devising strategies to overcome these challenges.^25–27^ The inclusion of even a single targeted question can provide unique insights into the influence of gender norms on health outcomes, especially when the question is balanced in seeking responses from gender-representative samples about what people of their own and other genders think. In previous research, attitudes reflected in response to the question, ‘Should young men/women wait to have sex until they are married?’ were juxtaposed with data-derived premarital sexual behaviour in Zambia to reveal varying degrees of discordance between attitudes and behaviours among adults—both men and women—which we coined the ‘taboo gap.’^28^ We found that the larger the regional-level taboo gap, the greater the risk for HIV infection among adolescent girls but not boys in those regions in Zambia.^3^

While missing information for women is the focus of much of the discourse on gender-related data, more attention to information for men and household elders will also aid in understanding the relative importance of different population groups in influencing (and upholding) normative behaviours, as well as being affected by them.^29^ Moreover, understanding community norms allows for more targeted design of gender-transformative programming and monitoring of interventions to improve health outcomes in the future.^30^

## Bias in construction of survey questions

Since its inception in 1981, the WVS has assessed people’s values around democracy, religion, gender equality and other value-laden topics. In evaluating the gender-related data collected for 60 countries from >85 000 adults in 2010–2014 (wave 6),^31^ we found patterns around question construction with the potential of biasing the inference we might draw from these data about gender norms. Attitude questions with gender bias built into the phrasing included leading statements such as ‘When a mother works for pay, the children suffer.’ Such statements are not neutral and imply a one-sided story (since the reverse question is not asked) for which disagreement is difficult to interpret. For example, respondents might think children suffer when both parents work, but this view is not captured. Additionally, variable interpretation of biased phrasing deters us from pooling or comparing data across countries or waves, thus limiting generalisability. Further, attitude questions that embed the terms wife or husband in the stem suggest the questions apply only to heterosexual unions and often imply a directionality in couple power dynamics, for example, that husbands should be principal income earners (‘If a woman earns more money than her husband, it’s almost certain to cause problems’). Critically, the imposition of gender-based expectations in question construction can perpetuate misunderstandings and impede our ability to monitor change in norms that are evolving.

These challenges are also evident in the DHS. In the late 1990s, new modules were implemented in the DHS on domestic violence and women’s empowerment, with a corresponding rise in gender-related health publications.^10^ Attitude questions on circumstances for which intimate partner violence perpetrated by men against their wives is justified are now deeply rooted in the literature and have been propagated to many other surveys, including the WVS. While these questions unlocked an important area of research, they discount the fact that boys, men, transgender people and other gender diverse individuals are also victims of domestic violence and that perpetrators of such violence may be of any gender.^32^ Additionally, these questions may further stigmatise and limit programmatic inclusion of all genders as reflected in data showing that abused boys are less likely than girls to seek help.^33^

Changing established questions impacts our ability to study response trends, and transforming survey questions into gender-neutral versions may present a challenge in strongly heteronormative contexts (eg, men may be reluctant to disclose experiences of intimate partner violence). However, qualitative or mixed-methods research will be important tools for overcoming such challenges. Importantly, removing or updating gender-biased questions could make room for new questions that are more inclusive of all genders and have greater utility in understanding how attitudes and practices impact health and well-being for all, and how these are evolving across contexts and over time. Further research and global stakeholder engagement are needed to systematically explore surveys like the DHS and WVS, to challenge assumptions underlying questions, identify instances of gender-related bias or inconsistency, and propose modifications to language to minimise the introduction of bias.

## Bias in survey translation and adaptation

Some gendered terms, such as ‘housewife,’ might change in form and meaning depending on the country/language for which the survey was adapted, introducing cross-cultural bias. In previous research,^3^ we selected two questions pertaining to employment in the WVS to investigate this issue: ‘Being a housewife is just as fulfilling as working for pay. Do you strongly agree, agree, disagree, or strongly disagree?’ and ‘Are you employed now or not?’ If not, response options were: ‘retired, housewife not otherwise employed, student, unemployed, other.’ We reviewed the questions in 48 surveys (26 languages and 37 countries, see table 1). The equivalent of ‘housewife’ was used in all cases when assessing the satisfaction this role provides. A gender-neutral option for employment was available in place of ‘housewife’ in only two countries (eg, do housework) while another three countries listed ‘housewife or househusband’ (or equivalent).

**Table 1:** English back-translation or local adaptation of ‘housewife by language, country and question asked in wave 6 of the World Value Survey^31^

Language	Country	V54: Do you strongly agree, agree, disagree or strongly disagree: Being a housewife is just as fulfilling as working for pay	V229: ‘are you employed or not?’ if not, response options were: ‘retired, housewife not otherwise employed, student, unemployed, other.’
Arabic	Armenia	Housewife	Housewife
Arabic	Kuwait, Qatar	Housewife	Housewife, not otherwise employed
Arabic	Libya	Housewife	Housewife/not employed
Azerbaijani	Azerbaijan	Housewife	Housewife, not otherwise employed
Chinese	Hong Kong	Housewife	Full-time taking care of family matters
English	Australia, South Africa	Housewife	Housewife
English	Cyprus, Ghana, Haiti, Nigeria, Singapore, Thailand, Trinidad and Tobago, Tunisia, Zimbabwe	Housewife	Housewife not otherwise employed
English	New Zealand	Housewife	Housewife/husband—home duties
Ewe	Ghana	Housewife	Housewife, not otherwise employed
Filipino	Philippines	Housewife	Housewife, not otherwise employed
French	Morocco	Housewife	Housewife or househusband
Ga	Ghana	Housewife	Housewife, not otherwise employed
German	Germany	Housewife	Housewife, househusband without any other occupation
Greek	Cyprus	Housewife	Housewife, not otherwise employed
Hausa	Ghana, Nigeria	Housewife	Housewife, not otherwise employed
Hindi	India	Housewife	Do housework
Igbo	Nigeria	Housewife	Housewife, not otherwise employed
Japanese	Japan	Housewife	Full-time housewife (don’t work at all)
Kinyarwanda	Rwanda	Housewife	Housewife, not working
Ndebele	Zimbabwe	Housewife	Housewife, not otherwise employed
Portuguese	Brazil	Housewife	Unpaid housewife
Romanian	Romania	Housewife	Housewife, not otherwise employed
Shona	Zimbabwe	Housewife	Housewife, not otherwise employed
Spanish	Argentina, Chile	Housewife	Housewife
Spanish	Colombia, Ecuador, Mexico, Peru, Uruguay	Housewife	Housewife who has no other job
Spanish	Spain	Housewife	Housewife/her chores
Swedish	Sweden	Housewife	Housewife
Taiwanese	Taiwan	Housewife	Housewife and no work
Turkish	Cyprus	Housewife	Housewife, not otherwise employed
Twi	Ghana	Housewife	Housewife, not otherwise employed
Urdu	Pakistan	Woman that stays at home	Stay at home woman who is not otherwise employed
Yoruba	Nigeria	Housewife	Housewife, not otherwise employed

Although intended as a term for those who manage a household, the phrasing of ‘housewife’ carries regionally varying connotations that can skew responses towards a societal view of the term rather than the intended purpose and create cross-regional differences reflective of the translation or adaptation of the question, confounding our understanding of the issues under investigation. Interestingly, evidence suggests that languages in which nouns are assigned a gender are associated with lower women’s labour force participation and perpetuate unequal treatment of women.^34^ Phrasing questions in gender-neutral terms across all languages and contexts is difficult, or in some cases, impossible. However, working with local language experts to systematically examine and address unintended bias in translation and adaptation of multicountry surveys is necessary to ensure their reliability and comparability.

## Conclusion

A 2016 United Nations report on the integration of gender statistics into national programmes^35^ details priorities in incorporating a gender perspective into surveys or censuses. They recommended >63 indicators critical for monitoring global progress toward gender equality by improving data for women, including sex-disaggregation, gender parity, reproductive health and gender-based violence, and policy-level normative data (eg, maternity leave). However, the recommendations are less helpful for elucidating underlying causes of gender disparities. None of the indicators address the conflation of gender and sex; missing information for men, boys and gender minorities; or biases in question construction, survey translation or adaptation.

While reasonable approaches exist and new approaches can be found for overcoming these shortcomings, limitations in resources and global stakeholder engagement will need resolution if we are to obtain and use improved data sources to advance our understanding of gender-based challenges to health and well-being for all. Most global surveys are designed in, and funded by, the global north and implemented in the global south, influencing not only who and what is measured but also risking the perpetuation of neocolonial biases and assumptions. The ‘decolonising global health’ movement argues for the need to restructure global health systems, move towards country ownership and equitable partnerships, and give voice to those with locally relevant and lived experience.^36 37^ We extend this need to the systems used to collect global health and gender-related data.

Integrating gender-related measures in global surveys is an opportunity to advance methods needed to disentangle contributions of sex-based differences from gender-related disparities in health. Lessons could be learnt from gender-related data collection in sectors such as agricultural or economics and applied to health. Use of qualitative gender-related data alongside survey data is important for gaining context-based insights into interpretation of quantitative findings. Importantly, global collaborations across data collection organisations—especially stakeholders in the global south—are needed to set standards for how we measure gender in all its forms in the future.

## Data Availability

Data derived from public domain resources.
